# Electrolysis in Stricture—Report of Three Cases with Remarks

**Published:** 1888-07

**Authors:** J. J. Berry

**Affiliations:** Portsmoth, N. H.


### ELECTROLYSIS IN STRICTURE—REPORT OF THREE CASES WITH REMARKS.

By J. J. Berry, M. D., Portsmoth, N. H.

Although my experience in the electrical treatment of urethral stricture has not been large, this report of three consecutive cases may prove useful in the collective investigation of the value of this method of treatment.

CASE I.—Strictures of four years standing, in patient thirty years of age, two in number. One two inches from meatus, admitting No. 10 American bougie. Another, five inches down, taking No. 6 only.

*Treatment.*—A No. 8 insulated, olive-pointed, Newman electrode was passed down to the second stricture and connected with a current of four cells. After an interval of five minutes, it was increased to five; at the end of twelve minutes, the instrument passed with little manipulation through the opening. He received no treatment whatever for a week, at the end of which time a No. 8 sound readily passed the obstruction. The subsequent treatment consisted of dilatation with sounds which were increased up to No. 18 American. No relapse.

CASE II.—Disease of six or seven years standing, and consisting of an obstruction four and a half inches from the meatus. It was found to admit a No. 10 bulb. In this case a No. 12 bulbous bougie of an older patent was employed. At the first attempt I was unable to pass it, but succeeded a few days afterwards. The electrode was not used again, the steel sound being employed exclusively. The patient, after being under observation two weeks, suddenly left the city and has never since been heard from. At date of last note, however, a No. 16 sound easily passed the stricture.

In both of these cases the stricture was of the annular variety with no great increase of connective tissue.

CASE III.—This patient had experienced difficulty in passing water for fifteen or twenty years with one or two attacks of retention. On examination, I was able to introduce a No. 13 sound about four and a half inches, at which place the canal suddenly narrowed down to a very small calibre and it was with extreme difficulty that I was able to pass a No. 4 whalebone into the bladder. This whole tract, involving an extent of three or more inches, appeared to be a dense mass of fibrous tissue, surrounding a very tortuous canal. Made several attempts to pass this stricture by electrolysis, but was never successful. The No. 7 electrode was used, with a current varying in strength from four to eight cells. I was able to enter the stricture a half inch but all efforts to go further failed. Yet, in spite of this, the patient declared that he could pass his water much better than before coming under treatment. Few will be inclined to deny the value of electrolysis in cases of this nature, yet like all other methods of treatment, it is not of universal application, much depends upon the skill and experience of the operator. With the common battery it is at times exceedingly difficult to estimate the exact strength of the current. The operation will often fail in those cases where there is an extensive deposit of inflammatory material. Yet the new tunnelled electrode, which may be passed down over a guide, will doubtless enable us at times to follow out a long and tortuous canal. The passage of a stricture by electrolysis enables one to practice subsequent dilatation with greater ease and success.

Thin, annular strictures seem to be specially adapted to this method, and I have no doubt would, without exception, yield to electricity. It is, however, worthy of trial in all cases, as the procedure is not only painless but devoid of all danger.

